# Survival prospects after acute myocardial infarction in the UK: a matched cohort study 1987–2011

**DOI:** 10.1136/bmjopen-2016-013570

**Published:** 2017-01-20

**Authors:** Lisanne A Gitsels, Elena Kulinskaya, Nicholas Steel

**Affiliations:** 1 School of Computing Sciences, University of East Anglia, Norwich Research Park, Norwich, UK; 2 Norwich Medical School, University of East Anglia, Norwich Research Park, Norwich, UK

**Keywords:** PRIMARY CARE, PREVENTIVE MEDICINE, All-cause mortality

## Abstract

**Objectives:**

Estimate survival after acute myocardial infarction (AMI) in the general population aged 60 and over and the effect of recommended treatments.

**Design:**

Cohort study in the UK with routinely collected data between January 1987 and March 2011.

**Setting:**

310 general practices that contributed to The Health Improvement Network (THIN) database.

**Participants:**

4 cohorts who reached the age of 60, 65, 70, or 75 years between 1987 and 2011 included 16 744, 43 528, 73 728, and 76 392 participants, respectively. Participants with a history of AMI were matched on sex, year of birth, and general practice to 3 controls each.

**Outcome measures:**

The hazard of all-cause mortality associated with AMI was calculated by a multilevel Cox's proportional hazards regression, adjusted for sex, year of birth, socioeconomic status, angina, heart failure, other cardiovascular conditions, chronic kidney disease, diabetes, hypertension, hypercholesterolaemia, alcohol consumption, body mass index, smoking status, coronary revascularisation, prescription of β-blockers, ACE inhibitors, calcium-channel blockers, aspirin, or statins, and general practice.

**Results:**

Compared with no history of AMI by age 60, 65, 70, or 75, having had 1 AMI was associated with an adjusted hazard of mortality of 1.80 (95% CI 1.60 to 2.02), 1.71 (1.59 to 1.84), 1.50 (1.42 to 1.59), or 1.45 (1.38 to 1.53), respectively, and having had multiple AMIs with a hazard of 1.92 (1.60 to 2.29), 1.87 (1.68 to 2.07), 1.66 (1.53 to 1.80), or 1.63 (1.51 to 1.76), respectively. Survival was better after statins (HR range across the 4 cohorts 0.74–0.81), β-blockers (0.79–0.85), or coronary revascularisation (in first 5 years) (0.72–0.80); unchanged after calcium-channel blockers (1.00–1.07); and worse after aspirin (1.05–1.10) or ACE inhibitors (1.10–1.25).

**Conclusions:**

The hazard of death after AMI is less than reported by previous studies, and standard treatments of aspirin or ACE inhibitors prescription may be of little benefit or even cause harm.

**Strengths and limitations of this study**

- Large cohort study representative of the full range of patients seen in routine clinical practice in the UK, which has a better coverage of acute myocardial infarction (AMI) patients than hospital records or disease registers.
- The matched study design allowed to estimate the effect of a history of AMI on all-cause mortality compared with no history of AMI while adjusting for a wide range of confounders.
- Although the major confounders of AMI were adjusted for, there could potentially be some residual confounding by indication for the treatments.

## Introduction

Survival after acute myocardial infarction (AMI) has improved over the past decades in Western countries including the UK both in the short and long term,^1–6^ partly due to an increase in coronary revascularisation, more effective drug therapy, and healthier lifestyles.^1–3^ ^6^ ^7^ The prevalence of AMI has increased, partly due to the ageing population, which makes evaluating long-term survival prospects increasingly important for setting out healthcare requirements and resource planning. Previous studies have estimated mortality rates of AMI standardised for age, sex, deprivation or region^2–6^ and examined survival variations in AMI patients, usually selected patients through hospitals or registries, by a range of confounders.^1^ ^2^ ^5^ ^7–12^ A recent population-based cohort study in England with data from 2004 to 2010 concluded that after 7 years people with a first or recurrent AMI had double or triple the risk of mortality compared with the general population of equivalent sex and age.^5^ These hazards are likely to be overestimated, because the study did not include controls and could therefore only compare the results with the sex-standardised and age-standardised mortality rates of the general population. AMI patients may be more likely to have comorbidities and an unhealthy lifestyle, which are independent predictors of survival, and so adjustment for these confounders is important.^13–15^

There is a need for a study that estimates long-term survival prospects after AMI, adjusts for important confounders, and assesses the impact of treatments on survival. With primary care data, information on demographics, lifestyle factors, comorbidities, and treatments is available for both cases and controls, thus allowing to estimate the adjusted survival difference between the two groups. Additionally, primary care has a better coverage of patients with AMI than hospitals and registers, because it includes patients who were diagnosed immediately and patients who were not sent to the hospital but were diagnosed in routine practice later by blood test results.^16^ Between 2003 and 2009, primary care covered 75% of the AMI cases in England while hospital and register data covered 68% and 52%, respectively.^16^ The three data sources had similar prevalence of risk factors and mortality rates of AMI.^16^

The objectives of this study were to estimate the hazard of mortality associated with a history of a single or multiple AMIs at key ages in UK residents while controlling for a wide range of confounders, and to estimate how survival prospects of AMI patients were changed by coronary revascularisation and recommended drug therapy.

## Methods

### Study design

This matched cohort study made use of medical records from The Health Improvement Network (THIN) database. These records are representative of the UK population regarding demographics, prevalence of medical conditions, and mortality rates when adjusted for deprivation.^17^ ^18^

Four cohorts of patients who were born between 1920 and 1940 and turned the initial age in 1987–2011 were selected. The initial ages were 60, 65, 70, and 75, chosen to provide advice on future management plans and resource planning at key ages.^14^ The selected patients had to be registered for at least 1 year at a general practice that coded death dates validly. The patient's record had to include a postcode and should have been accessed at least once within the past 10 years. From these cohorts, patients with a history of AMI were selected and each was matched to three controls without history on sex, year of birth category, and general practice. The study's end date was the 18th of March 2011, thus patients were followed-up for up to 24 years. Patients could be part of multiple cohorts. Patients who changed general practice during the study could no longer be observed. It was assumed that the loss to follow-up was not associated with the outcome mortality.

### Patient involvement

No patient was involved in setting the research question, outcome measures, design or conduct of the study. The results were not disseminated to the patients, as the study was based on anonymised patient records.

### Variable selection

The baseline characteristics of patients were assessed on the 1st of January of the year they turned the cohort's age. The primary exposure was AMI. Multiple events were required to be separated by 30 days. Information on the type of AMI was not available. However, a study that linked information from the Myocardial Ischaemia National Audit Project (MINAP) and the General Practice Research Database (GPRD), which has 60% of practices in overlap with THIN, found that 46% of AMIs were ST-elevated (ST segment elevation myocardial infarctions, STEMIs) in England and Wales in 2003–2008.^19^ The selected confounders were based on literature review, and consisted of: sex, year of birth, socioeconomic status, angina pectoris, heart failure, other cardiovascular conditions (valvular heart disease, peripheral vascular disease, and cerebrovascular disease), chronic kidney disease, diabetes mellitus, hypertension, hypercholesterolaemia, alcohol consumption, body mass index (BMI), and smoking status (see online supplementary tables SA1 and SA2). Socioeconomic status was measured by Mosaic, which is based on demographics, lifestyles, and behaviour of people at a postcode level.^20^

10.1136/bmjopen-2016-013570.supp1 supplementary material

The treatment investigated was based on the UK National Institute of Health and Care Excellence (NICE) recommended first-line treatment to AMI patients during the study period, which includes: coronary revascularisation and prescription of ACE inhibitors, aspirin, β-blockers, calcium-channel blockers, and statins.^21–23^ Since 2007, calcium-channel blockers are only recommended to treat hypertension or angina in AMI patients.^22^ ^23^ Since 2013, dual antiplatelet therapy (DAPT: aspirin plus another antiplatelet agent) are recommend to AMI patients.^22^ ^23^ Owing to the low prevalence of DAPT in the age cohorts, the survival effect of the therapy were not estimated (see online supplementary table SA3). Family history of AMI or cardiovascular disease were not included in the analysis because of the very low rates of recording in primary care.^24^ Indicators of psychosocial factors such as job strain and lack of social support, fruit and vegetable intake, and physical activity were not included in the analysis because THIN does not hold information on them.

There were missing values in alcohol consumption (proportion range across the four cohorts 17–37%), BMI (18–37%), and smoking status (10–29%). The fraction of incomplete medical records decreased with age; 45% of the youngest cohort and 23% of the oldest cohort had incomplete records. Incomplete records were more common in patients born at an earlier year and in patients without medical conditions or on treatments (see online supplementary table SA4). This is in accordance with previous research that reported that recording has improved since the introduction of Quality and Outcomes Framework (QOF) in 2004.^25–27^ Missing values were dealt with by multiple imputation.^28^ The distribution of known and imputed values were similar (see online supplementary table SA5).

### Statistical analyses

A Cox's proportional hazards regression model was fitted to estimate the effect of a history of AMI and respective treatments on the hazard of all-cause mortality at different ages. The outcome variable was time to death in days, that is, from 1st of January of the year the patient turned the cohort's age to the date of death. Starting from a model with second-order interaction effects of all variables with the main exposure AMI and the matching factors sex and year of birth, backward elimination was performed to obtain the most parsimonious model possible. Interaction effects found in the complete case analysis, that is, the analysis that excluded patients with incomplete medical records, which were not restricted to the main exposure and matching factors, were also included in the backward elimination process. A unified model for all ages was chosen to have the same interpretation of the hazards.

The final model included sex, year of birth, socioeconomic status, AMI, angina, heart failure, other cardiovascular conditions, chronic kidney disease, diabetes, hypertension, hypercholesterolaemia, coronary revascularisation, β-blockers, ACE inhibitors, calcium-channel blockers, aspirin, statins, alcohol consumption, BMI, smoking status, general practice, and interactions of AMI with angina, AMI with β-blockers, AMI with calcium-channel blockers, hypercholesterolaemia with statins, and BMI with smoking status. Chronic kidney disease was not adjusted for at ages 60 and 65 due to low prevalence of <1%.

The number of years gained or lost due to a history of AMI, coronary revascularisation, and drug therapy were calculated.^29^ The models were assessed on validity of proportional hazards assumption, overall performance, discrimination, and external validation.^30–32^ The sensitivity analysis compared the unadjusted and adjusted effect of a history of AMI estimated on the imputed datasets.

For more detailed information on the statistical analyses, please see online supplementary data.

## Results

**Figure 1:**
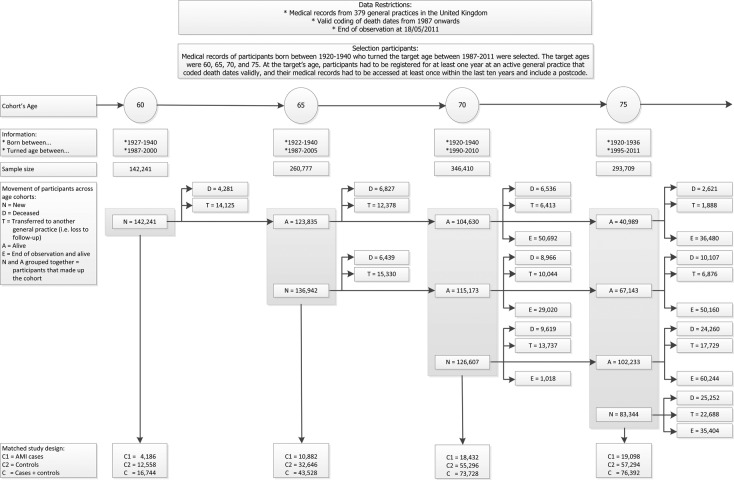
Selection of cohorts. AMI, acute myocardial infarction.

The prevalence of comorbidities was higher among AMI cases than controls (figure 1 and table 1). Obesity (BMI≥30 kg/m^2^) was more common among cases, whereas overweight (BMI 25–30 kg/m^2^) was as common among cases as controls. The prevalence of smokers was the same in the two groups, while the prevalence of ex-smokers was greater among cases.

**Table 1:** Characteristics of acute myocardial infarction (AMI) cases and controls by age cohort

	Age 60		Age 65		Age 70		Age 75	
	Cases*	Controls†	Cases	Controls	Cases	Controls	Cases	Controls
Number of participants	4186	12 558	10 882	32 646	18 432	55 296	19 098	57 294
Total person-years of follow-up (mean)	46 686 (11.2)	150 471 (12.0)	93 056 (8.6)	299 841 (9.2)	114 700 (6.2)	370 006 (6.7)	91 884 (4.8)	298 140 (5.2)
Deaths during follow-up (%)	1220 (29%)	2008 (16%)	3070 (28%)	5782 (18%)	5186 (28%)	10 557 (19%)	5895 (31%)	12 674 (22%)
Transferred during follow-up (%)	900 (22%)	3035 (24%)	1986 (18%)	6597 (20%)	2693 (15%)	8781 (16%)	2733 (14%)	8971 (16%)
Male (%)	3367 (80%)	10 101 (80%)	8402 (77%)	25 206 (77%)	13 567 (74%)	40 701 (74%)	13 163 (69%)	39 489 (69%)
Angina (%)	1924 (46%)	594 (5%)	5161 (47%)	2445 (7%)	8623 (47%)	5528 (10%)	9122 (48%)	7472 (13%)
Heart failure (%)	205 (5%)	61 (0%)	676 (6%)	338 (1%)	1568 (9%)	982 (2%)	2198 (12%)	1674 (3%)
Other cardiovascular conditions (%)	979 (23%)	681 (5%)	3154 (29%)	2941 (9%)	6591 (36%)	7672 (14%)	8205 (43%)	11 674 (20%)
Chronic kidney disease (%)	1 (0%)	0 (0%)	4 (0%)	8 (0%)	965 (5%)	1392 (3%)	1872 (10%)	3039 (5%)
Diabetes (%)	449 (11%)	624 (5%)	1622 (15%)	2297 (7%)	3398 (18%)	5573 (10%)	3726 (20%)	6876 (12%)
Hypercholesterolaemia (%)	1634 (39%)	1907 (15%)	4228 (39%)	7423 (23%)	6392 (35%)	14 936 (27%)	6395 (33%)	15 814 (28%)
Hypertension (%)	1168 (28%)	1991 (16%)	3750 (34%)	7608 (23%)	7411 (40%)	17 955 (32%)	8579 (45%)	22 330 (39%)
Alcohol consumer (%)‡	3385 (81%)	10 997 (88%)	8780 (81%)	28 130 (86%)	14 494 (79%)	45 962 (83%)	14 293 (75%)	45 504 (79%)
Overweight (%)‡	2427 (58%)	7239 (58%)	5866 (54%)	17 609 (54%)	9406 (51%)	28 253 (51%)	9264 (49%)	28 030 (49%)
Obese (%)‡	750 (18%)	1418 (11%)	2295 (21%)	4687 (14%)	4107 (22%)	9180 (17%)	3848 (20%)	9365 (16%)
Ex-smoker (%)‡	1274 (30%)	2398 (19%)	4611 (42%)	10 903 (33%)	8335 (45%)	19 305 (35%)	8695 (46%)	20 641 (36%)
Smoker (%)‡	1163 (28%)	3507 (28%)	2203 (20%)	6544 (20%)	3079 (17%)	8973 (16%)	2545 (13%)	7660 (13%)

*Participants with a history of AMI.

†Participants with no history of AMI.

‡Mean 10 imputed datasets.

### Prevalence of treatment

The prevalence of coronary revascularisation and drug therapy was higher among patients who had multiple AMIs compared with patients who had a single AMI (table 2). The rates across the four age cohorts for coronary artery bypass graft (CABG) and percutaneous coronary intervention (PCI) were 16–19% and 3–8%, respectively (see online supplementary table SA6). Men were approximately twice as likely to have had coronary revascularisation as women were, which could not be explained by age, deprivation, or diabetes (see online supplementary figure SA1 and supplementary figure SA2). The difference in treatment prevalence by the four initial ages converged over time. In 2010 the most widely prescribed drugs to AMI patients were statins (94%) and aspirin (94%) followed by ACE inhibitors (85%), β-blockers (65%), and calcium-channel blockers (25%). In the same year, 38% of the AMI patients have had coronary revascularisation by an initial age; the prevalence was greater in patients living in the most affluent areas (index of multiple deprivation (IMD) category 1: 45%) than in patients living in the most deprived areas (IMD category 5: 32%), trend χ^2^ (1)=5.06, p=0.02.

**Table 2:** Baseline treatment given a possible history of IHD

			Coronary revascularisation		Drug therapy				
Cohort*	IHD	Size	Men	Women	Aspirin	ACE inhibitors	β-blockers	Statins	Ca-channel blockers†
Age 60	No	11 964	0 (0%)	0 (0%)	271 (2%)	678 (6%)	1156 (10%)	208 (2%)	615 (5%)
	Angina	594	97 (19%)	7 (8%)	211 (36%)	95 (16%)	238 (40%)	148 (25%)	264 (44%)
	Single AMI	3465	486 (18%)	77 (11%)	1467 (42%)	768 (22%)	1482 (43%)	951 (27%)	1080 (31%)
	Multiple AMIs	721	194 (32%)	20 (18%)	386 (54%)	256 (36%)	314 (44%)	247 (34%)	290 (40%)
Age 65	No	30 201	0 (0%)	0 (0%)	2548 (8%)	3299 (11%)	3727 (12%)	2194 (7%)	2709 (9%)
	Angina	2445	512 (25%)	30 (7%)	1400 (57%)	701 (29%)	1036 (42%)	1164 (48%)	1024 (42%)
	Single AMI	8796	1532 (23%)	334 (16%)	5751 (65%)	3452 (39%)	4011 (46%)	4722 (54%)	2762 (31%)
	Multiple AMIs	2086	594 (35%)	67 (17%)	1532 (73%)	1072 (51%)	946 (45%)	1272 (61%)	722 (35%)
Age 70	No	49 768	0 (0%)	0 (0%)	8698 (17%)	9756 (20%)	7176 (14%)	8863 (18%)	6820 (14%)
	Angina	5528	1263 (28%)	125 (12%)	3851 (70%)	2204 (40%)	2376 (43%)	3335 (60%)	2235 (40%)
	Single AMI	14 847	2811 (26%)	730 (18%)	11 269 (76%)	7770 (52%)	6989 (47%)	9638 (65%)	4461 (30%)
	Multiple AMIs	3585	1012 (36%)	172 (22%)	2918 (81%)	2202 (61%)	1721 (48%)	2524 (70%)	1219 (34%)
Age 75	No	49 822	0 (0%)	0 (0%)	12 592 (25%)	12 633 (25%)	7945 (16%)	11 318 (23%)	8574 (17%)
	Angina	7472	1652 (29%)	225 (13%)	5642 (76%)	3430 (46%)	3188 (43%)	4780 (64%)	2952 (40%)
	Single AMI	15 319	2705 (26%)	835 (17%)	12 487 (82%)	9226 (60%)	7036 (46%)	10 395 (68%)	4676 (31%)
	Multiple AMIs	3779	954 (35%)	230 (23%)	3295 (87%)	2574 (68%)	1759 (47%)	2767 (73%)	1228 (32%)

*The age cohorts included cases with history of AMI who were matched to three controls on sex, year of birth category, and general practice. The prevalence of treatment by the initial ages was affected by calendar year (see online supplementary figure SA2).

†First-line drugs prescription until 2007 after which it became a second-line drugs prescription.^2^

AMI, acute myocardial infarction; IHD, ischaemic heart disease.

### Survival prospects after AMI

**Figure 2:**
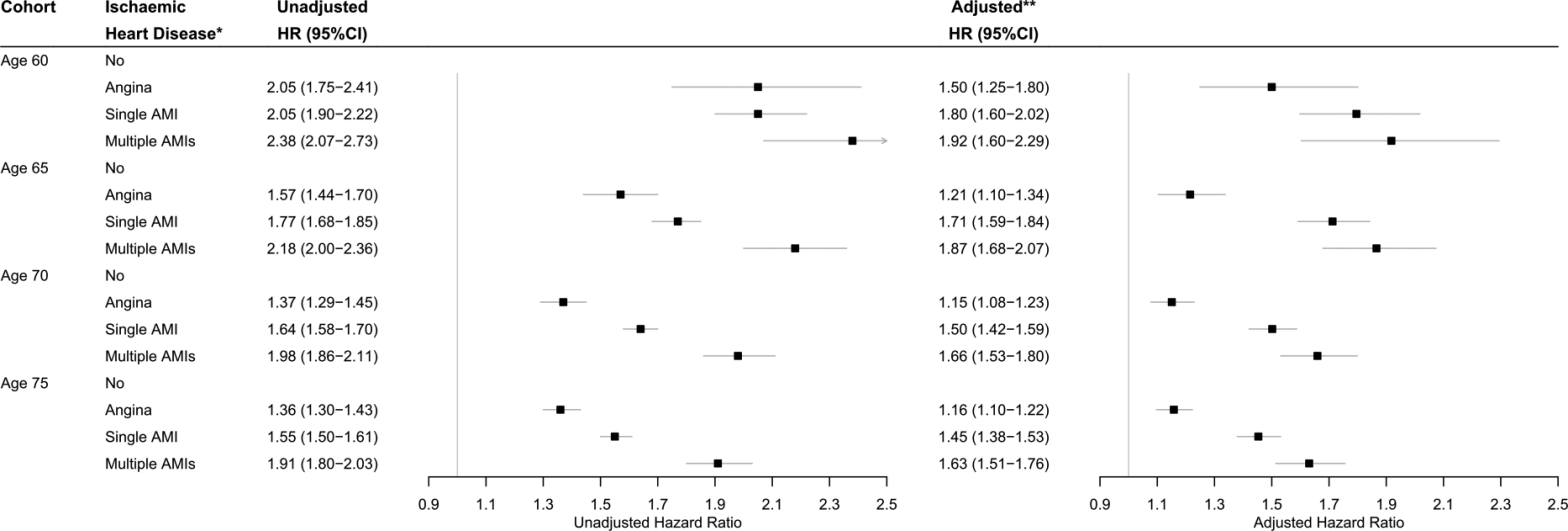
Unadjusted and adjusted effects of a history of ischaemic heart disease on the hazard of all-cause mortality. *Age cohorts consisted of cases who had a history of acute myocardial infarction (AMI) and controls who had no history of AMI. The hazard of mortality associated with single/multiple AMIs includes possible history of angina. **Adjusted for sex, year of birth, socioeconomic status, heart failure, other cardiovascular conditions, chronic kidney disease (only at ages 70 and 75), diabetes, hypertension, hypercholesterolaemia, coronary revascularisation, statins, β-blockers, ACE inhibitors, calcium-channel blockers, aspirin, alcohol consumption, body mass index, smoking status, and general practice.

The adjusted hazard of all-cause mortality for AMI patients was constant during follow-up of 24 years; it did not matter how many years the cases had already survived, they were still at a higher risk of dying than the controls. This relative risk was the greatest in the youngest cohort while the absolute risk was the greatest in the oldest cohort (figure 2 and see online supplementary figure SA3). Compared with no history of AMI by age 60, 65, 70, or 75, having had one AMI was associated with an adjusted hazard of mortality of 1.8 (1.6 to 2.0), 1.7 (1.6 to 1.8), 1.5 (1.4 to 1.6), or 1.5 (1.4 to 1.5), respectively. This translates to a decrease in life expectancy of 5.9 (4.7 to 7.0), 5.4 (4.6 to 6.1), 4.1 (3.5 to 4.6), and 3.7 (3.2 to 4.3) years, respectively. Compared with no history of AMI by age 60, 65, 70, or 75, having had multiple AMIs was associated with an adjusted hazard of mortality of 1.9 (1.6 to 2.3), 1.9 (1.7 to 2.0), 1.66 (1.5 to 1.8), or 1.6 (1.5 to 1.8), respectively. This translates to a decrease in life expectancy of 6.5 (4.7 to 8.3), 6.2 (5.2 to 7.3), 5.1 (4.3 to 5.9), or 4.9 (4.1 to 5.6) years, respectively. The hazard of mortality did not differ between cases with or without a history of angina. There were also interactions with prescriptions of β-blockers and calcium-channel blockers, which are described below. There were no other interactions with a history of AMI, meaning that the effect of AMI on the hazard of mortality was the same for different groups of patients, such as for men and women. The comorbidities that had the greatest impact on survival were other cardiovascular conditions and heart failure (see online supplementary figures SA4–7). The associated impact was greatest in the youngest age cohort. On average the comorbidities led to an additional decrease in life expectancy of 4.6–7.1 years.

**Figure 3:**
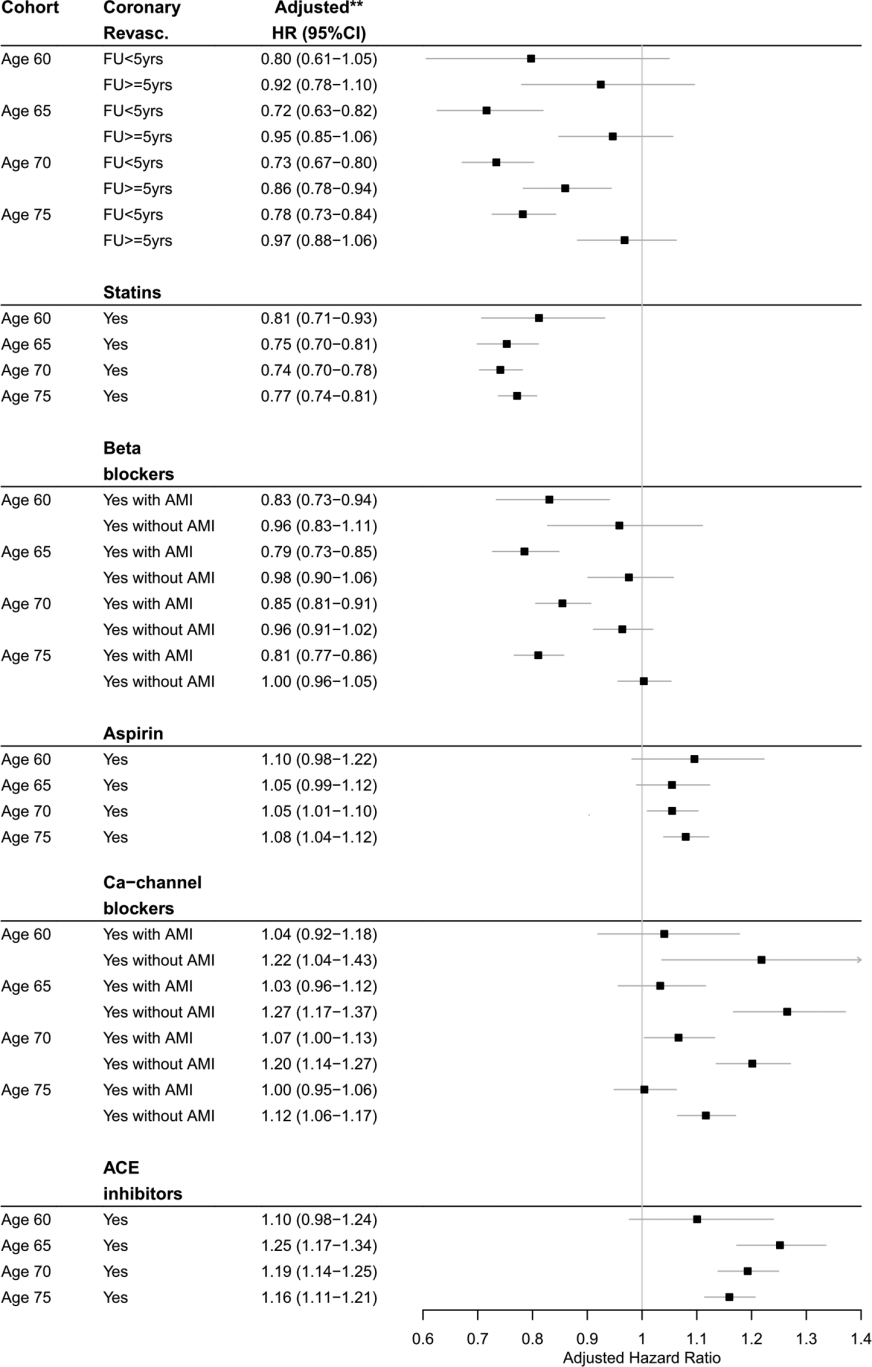
Adjusted effects of a history of treatment on the hazard of all-cause mortality. *Time-varying effect of a history of coronary revascularisation on the hazard of mortality was split at 5 years of FU after the initial age. **Adjusted for sex, year of birth, socioeconomic status, AMI, angina, heart failure, other cardiovascular conditions, chronic kidney disease (only at ages 70 and 75), diabetes, hypertension, hypercholesterolaemia, alcohol consumption, body mass index, smoking status, general practice, and listed treatments. Results of β-blockers and calcium-channel blockers are reported separately for cases and controls, because there was an interaction effect. AMI, acute myocardial infarction; Ca-channel, calcium-channel; FU, follow-up; revasc., revascularisation; yrs, years.

Coronary revascularisation was associated with a significant improvement in the survival prospects in the short-term (figure 3). Compared with no history of coronary revascularisation by age 60, 65, 70, or 75, having had revascularisation was associated with an adjusted hazard of mortality of 0.8 (0.6 to 1.1), 0.7 (0.6 to 0.8), 0.7 (0.7 to 0.8), and 0.8 (0.7 to 0.8), respectively, in the first 5 years of follow-up. This translates to an increase in life expectancy of 2.3 (−0.5 to 5.0), 3.3 (2.0 to 4.7), 3.1 (2.2 to 4.0), and 2.5 (1.7 to 3.2) years, respectively. After 5 years of follow-up, a history of coronary revascularisation was no longer associated with a significant improvement in the survival prospects. These prospects were the same for men and women.

Drug therapy was associated with mixed survival prospects and could differ by subgroups of patients (figure 3). The drug therapy that was associated with the greatest improved survival prospects was prescription of statins; the prescription translated to an average increase in life expectancy of 2.5 years at all ages. The hazard of mortality associated with statins prescription did not differ between patients with or without a history of hypercholesterolaemia. Prescription of β-blockers was associated with mixed survival prospects; prescription translated to an average increase in life expectancy of 2.0 years at all ages in AMI patients versus no increase in patients without AMI. Prescription of calcium-channel blockers was also associated with mixed survival prospects; prescription translated to no increase in life expectancy in AMI patients versus an average decrease in life expectancy of 2.0 years in patients without AMI. Prescription of aspirin or ACE inhibitors was associated with worsened survival prospects; the prescription translated to an average decrease in life expectancy of 1.0 and 1.5 years, respectively, at all ages. There were no significant differences in the effects of the treatments by sex.

Survival prospects differed by socioeconomic status, in which the difference was greater at a younger age. The Mosaic category 5 (‘neighbourhood with mainly young couples’) was associated with the worst survival prospects for patients aged 60 and older, this ranged from an adjusted hazard of mortality of 1.7 (1.4 to 2.1) at age 60 to 1.3 (1.2 to 1.4) at age 75 (see online supplementary figures SA3–6). In addition, survival prospects varied considerably between general practices. The 95% tolerance interval of the adjusted hazard of mortality associated with general practice was 0.8 to 1.2 at age 60 and 0.6 to 1.5 at older age. This translates to an average of 4.5 and 10.0 years difference in life expectancy, respectively. A general practice could serve a range of patients with regards to health status, ethnic background, deprivation, urbanisation, and pollution. These factors, however, did not explain the hazard of mortality associated with general practice (see online supplementary methods and table SA8).

### Model performance

Please see the online supplementary data for model performance and sensitivity analysis.

## Discussion

This matched cohort study estimated the adjusted hazard of all-cause mortality associated with a history of AMI and respective treatments by age 60, 65, 70, or 75 in UK residents using medical records from primary care between 1987 and 2011. In accordance with the literature, this study found that AMI survivors have a long term, increased hazard of mortality, in which younger survivors and survivors of multiple events were worse off.^1^ ^2^ ^5^ ^7–12^ However, this study estimated lower hazards of mortality than previously estimated. Survival was better in those who had coronary revascularisation or were prescribed statins or β-blockers, but worse in those prescribed aspirin or ACE inhibitors, and unchanged in those prescribed calcium-channel blockers. The estimated hazards of mortality associated with these treatments were almost the same at each age, implying that the effectiveness of treatments does not differ by age.

The lower estimated hazards of mortality associated with a history of AMI reported by this study compared with previous studies could be due to the different data source used and the range of confounders adjusted for. This study made use of primary care data, whereas most studies used hospital and register data. Research showed that the 1-year mortality rate of AMI is lower in primary care probably because of a lower proportion of severe cases.^16^ Furthermore, this study adjusted for a range of confounders which attenuated the estimated hazards of mortality associated with a history of AMI. There is a smaller difference between the unadjusted estimates of this study and the age-standardised and sex-standardised mortality ratios estimated in English residents based on hospital and register data from 2004 to 2010 by Smolina *et al*.^5^ It is unlikely that the lower estimated hazards of mortality reported by this study are due to the shifting epidemiological trends in cardiovascular disease because there were no interactions between a history of AMI and year of birth category or other risk factors with the exception of angina, β-blockers, and calcium-channel blockers. The medical advances and shifting prevalence of risk factors over time were adjusted for in the analysis and had no different survival effects in AMI patients compared with patients without AMI. This study did not find sex difference in survival prospects after AMI. This is supported by some studies^8^ ^10^ ^33^ ^34^ but contradicted by another.^5^ The difference could be explained by (the lack of) adjustment for comorbidities and treatments.^8^ ^10^ ^33^ ^34^

This study found that the lower uptake of coronary revascularisation by women could not be explained by age, diabetes, or deprivation, as suggested by a previous study.^10^ A study with data from the UK from 2003 to 2008 showed that coronary revascularisation was more prevalent in non-STEMIs than in STEMIs.^19^ As non-STEMIs are more common among women than among men,^19^ it seems that type of AMI could not explain the sex difference in uptake of surgery present in this study. In 2012, the European Society for Cardiology reviewed the sex differences in treatment after AMI, taking into account sex differences in risk profiles, and concluded that sex differences exist.^35^ This study also found that a history of coronary revascularisation was no longer associated with a significantly improved survival prospects after 5 years of follow-up. This is in accordance with another study that reported a protective effect in the 1-year mortality rate but an insignificant effect in the 5-year mortality rate of AMI.^10^ The findings suggest that coronary revascularisation might mainly be beneficial in reducing early mortality. No sex difference in survival after coronary revascularisation was found in this study, which is supported by some studies^5^ ^10^ but contradicted by another.^36^ This study found no difference in drugs prescriptions by sex by 2010, suggesting that the difference converged over time.^3^

The findings of this study agree with the clinical evidence reviewed by NICE^23^ on the effectiveness of statins and calcium-channel blockers, but disagree with the effectiveness of ACE inhibitors, aspirin, and β-blockers. The NICE review on ACE inhibitors estimated a protective effect in AMI patients with left ventricular systolic dysfunction (LVSD) and an inconclusive harmful effect in AMI patients with unselected LVSD in 1986–1993.^37–41^ Other studies not yet reviewed by NICE, estimated hazardous effects associated with ACE inhibitors and suggested that the results could be due to confounding by heart failure or indication and use of old data (1984–2005).^1^ ^12^ ^42^ The current study controlled for heart failure, which lowered the HR of ACE inhibitors by ∼0.05, and made used of more recent data from 1987 to 2011, thereby suggesting that ACE inhibitors might in fact be harmful to survival. The NICE review on aspirin only included one study that estimated an inconclusive protective effect of the drug versus placebo on all-cause mortality.^23^ That study included men with a recent AMI aged 30–64 in 1972–1974.^43^ The current study made use of more recent data with longer follow-up of older patients of both sexes. Aspirin is associated with an increased risk of bleeding, where the risk increases with age.^23^ Since the elderly are excluded from most clinical trials, it could be that aspirin might actually be harmful in the elderly as the findings of the current study suggest. The findings on β-blockers are in concordance with more recent published clinical studies^1^ ^11^ ^42^ that were not yet reviewed by NICE.

Finally, this study found that survival prospects varied greatly across general practices, which was independent from health status, ethnic background, deprivation, urbanisation, and pollution. Other studies have not reported survival variations by general practice, although it was adjusted for in a study by Gerber *et al*.^9^ That study estimated the effect of neighbourhood and individual socioeconomic status on survival after AMI and suggested that higher level measured socioeconomic status might capture residual confounding of unequal hospital resources and social characteristics of an area such as social cohesion and attitudes towards health.^9^

### Study's strengths and limitations

This study used routinely collected primary care data that were representative of the UK.^17^ ^18^ The advantage of using primary care data was that there was more information on sociodemographic and lifestyle factors available and there was a higher coverage of AMI cases.^16^ The matched study design allowed to estimate the effect of a history of AMI on mortality compared with no history of AMI while adjusting for a wide range of confounders. The confounders included comorbidities, treatments, lifestyle choices, and demographics, and interactions between these factors. This has not been done before; previous studies were either population-based which has a tendency to overestimate the hazardous effect of AMI on survival, or previous studies only included AMI cases which meant that only survival variations among AMI survivors could be estimated. Estimating the effect of a history of AMI at different ages meant that the results could be used for planning ongoing medical management and planning resources allocation in the British population. Finally, the study had a long follow-up of almost 25 years.

Data on the type of AMI were not available in THIN, therefore this study could not distinguish between STEMI and non-STEMI and thus could not provide specific survival prospects for them. Although the major confounders of AMI were adjusted for, there could potentially be some residual confounding by a number of other factors: family history of AMI or cardiovascular disease, psychosocial factors, fruit and vegetable intake, and physical activity. These factors were not adjusted for in the survival models due to the unsystematic or no recording in the medical records. AMI severity indicators, such as left ventricular function, were also not included in the survival models because this information was only available for the cases and not the controls. Missing data in lifestyle factors were dealt with by multiple imputations. This is a widely accepted method to deal with bias and imprecision when missing data are present.^28^ Adherence to drug therapy was unknown and therefore the survival prospects associated with prescription of drug therapy might not accurately reflect the effect of the drugs themselves on mortality. Furthermore, no dose–response effect could be estimated as the prescribed doses were not included in the survival models. Finally, there might be bias by indication in which patients receiving treatment were somehow sicker than those not receiving the treatment, despite the adjustment for important confounders.

### Recommendations

The findings of this study suggest that surviving an AMI is associated with a permanent increased hazard of mortality and that coronary revascularisation, statins prescription, and β-blockers prescription can reduce this hazard. This is of clinical importance, because not every AMI survivor receives these treatments. In 2010, β-blockers were not widely prescribed to AMI survivors; the survival prospects of 35% of the AMI survivors might be improved by such a prescription. This study suggested that there were sex and deprivation inequalities in uptake of coronary revascularisation while all subgroups benefitted equally from it.

This study also found that the prescription of aspirin and/or ACE inhibitors was associated with an increased hazard of mortality. This might be of potential concern as the previous explanations for similar findings on the hazardous effects associated with ACE inhibitors on survival, such as confounding by heart failure and use of old data, were addressed by this study. By 2010, 94% and 85% of AMI survivors were prescribed aspirin and ACE inhibitor, respectively. Further research is required to assess the effectiveness of aspirin and ACE inhibitors in the light of our findings that such commonly used medications may be of little benefit, or even cause harm.

Further research is needed to explore the reasons for the considerable unexplained survival variations between general practices.
