# Supplementary material for: Survival prospects after acute myocardial infarction in the UK: a matched cohort study 1987–2011
Source: BMJ Open. 2017 Jan 20;7(1):e013570. doi: 10.1136/bmjopen-2016-013570 (PMC5278246; doi:10.1136/bmjopen-2016-013570)
Supplement: supplementary material [file bmjopen-2016-013570supp.pdf]

SUPPLEMENTAL MATERIAL

Supplemental Methods

The study design was a matched cohort study, where patients with a history of AMI were selected and each matched to three controls without this history on sex, year of birth category, and general practice. The rationale behind matching cases to controls, was to create a more balanced dataset for the exposure was a relatively rare event. A more balanced dataset translates in increased statistical power and analytical efficiency.[1]

The analysis of the age cohorts was performed in two stages, where the first stage of complete case analysis informed the second stage of full data analysis. Frequency matching was carried out once on complete medical records and once irrespective of completeness of records. The reason for this was to ensure that the balance in the cohorts created by matching would not be undone when excluding patients with missing data for the complete case analysis.[1] Moreover, this led to optimal use of the available data by maximising the sample size of the complete case analysis and thereby increasing the statistical power. Patients could be part of multiple cohorts, where cases could be matched to different controls. Matching was done in Python version 3.4.2.

Incomplete medical records were dealt with by multiple imputation. Values were imputed for alcohol consumption, body mass index, and smoking status. The joint imputation model consisted of all factors from the snapshot of medical history including time to death and was multilevel to adjust for the correlation between patients from the same general practice. Imputations were done in REALCOM-Imputation software. The Monte Carlo Markov Chain (MCMC) estimation had a burn-in length of 100 iterations and was in total 1,000 iterations long. The imputed values of every 100th iteration were used. This resulted in ten imputed datasets. The effect of a history of AMI on survival were estimated on the imputed datasets and pooled by applying Rubin's rules to deal with the uncertainty caused by missing data.[2] Time-tied deaths in the Cox's proportional hazard regression were handled by Efron's approximation.[3]

A unified survival model for all ages was chosen to have the same interpretation of the hazards. The survival model consisted of the factors that were found significant in the majority of models; $p<.05$ for fixed effects and $p<.01$ for interaction effects. The model also included a random effect of general practice to adjust for the correlation between patients from the same practice.

Since we could not include postcode indicators in the original models because they were only recorded for patients from England and Wales, an additional analysis was carried out to examine the hazard of mortality associated with general practice in more detail for these patients. The additional confounders explored were: index of multiple deprivation (IMD), level of urbanisation, quintile of limiting long-term illness, quintile of various ethnic groups, quintile of air pollution, and percentage of AMI cases by initial age in the general practice (Table A1 in the Online Data Supplements). This information was derived from census data of England and Wales, and was thus not available for patients from Scotland or Northern Ireland. Even though these variables were measured on district level or higher, the relation between them and general practice was examined on patient level. This was because a practice could serve patients from a range of districts and an average calculated in the cohorts would not be representative of the practice as it would be skewed to the older and sicker patients. High density scatterplots were made and Spearman's rank tests were performed to estimate the correlation between the hazard of mortality associated with general practice and the variables.

The number of years gained or lost due to a history of AMI, coronary revascularisation, and drug therapy were calculated by multiplying the natural logarithm of the hazard ratio by ten.[4] This calculation is valid provided that the proportional hazards assumption is satisfied and the cumulative baseline hazard increases exponentially with age. The assumption of proportional hazards was tested by including an interaction term between the fixed effects and follow-up time.[5] When the interaction was significant ($p<.01$), the factor's effect on survival time was made time-variant. The assumption of the baseline hazard increasing exponentially with age holds when the logarithm of the baseline hazard against follow-up time results in a horizontal line.

The survival models were assessed on overall performance, discrimination, and external validation, using Royston's R^2, Harrell's concordance, and the shrinkage slope, respectively. Royston's R^2 computes the percentage explained variation in survival time by the model.[6] Harrell's concordance calculates the percentage of correspondence between the estimated hazard score and observed survival time for all combinations of two selected patients.[7] Survival models have usually a concordance between 60 and 70%. The shrinkage slope is the factor by which the regression coefficients need to be shrunk due to overfitting of the model. This was computed by a ten-fold cross-validation.[8] The sensitivity analysis compared the unadjusted and adjusted effect of a history of AMI estimated on the imputed datasets.

The analyses were performed in R version 3.1.1[9], using the packages 'rms'[8] and 'survival'[3].

Supplemental Results

Model performance

The adjusted survival models explained 20 to 29% of survival differentials. There was 68 to 70% concordance between the estimated hazard of mortality and survival time. The shrinkage slopes indicated that the adjusted effects were overestimated by less than 3%. The survival models that only included history of IHD explained less than 1% of survival differentials and had between 56-59% concordance between the estimated hazard of mortality and survival time. The difference between the unadjusted and adjusted estimates and the respective model performances demonstrate the importance of controlling for confounders when estimating the effect of a medical condition and treatments on the hazard of mortality.

Supplemental Tables & Figures

Table A1 Description and coding of variables

	**Description***	**Coding†**
Medical condition	Acute myocardial infarction (AMI) diagnosis, multiple AMIs had to have at least 30 days between events	No/single/multiple
	Angina pectoris diagnosis	No/yes
	Chronic kidney disease (CKD) stages 3-5 diagnosis (GFR<60mL/min)	No/yes
	Diabetes mellitus diagnosis	No/yes
	Heart failure diagnosis	No/yes
	Hypercholesterolaemia (HCL) diagnosis or a total cholesterol reading of greater than 5mmol/L	No/yes
	Hypertension diagnosis	No/yes
	Ischaemic heart disease (IHD), which include diagnosis of: angina pectoris, acute myocardial infarction, and subsequent events or complications of these conditions	No/angina/single AMI and possible angina/multiple AMIs and possible angina
	Other cardiovascular conditions (CVS), which include diagnosis of: valvular heart disease, peripheral vascular disease, cerebrovascular disease, and other cardiovascular system conditions	No/yes
Treatment	ACE inhibitor prescription (BNF chapter 2.5.5.1 and 2.5.5.2), which include: angiotensin-converting enzyme inhibitors and angiotensin-II receptor antagonists	No/yes
	Aspirin prescription (BNF chapter 2.9.1)	No/yes
	Beta-adrenoceptor blocking drugs prescription (BNF chapter 2.4)	No/yes
	Calcium-channel blocker prescription (BNF chapter 2.6.2)	No/yes
	Coronary revascularisation, which include coronary artery bypass graft (CABG) and percutaneous coronary intervention (PCI)	No/yes
	Statin prescription (BNF chapter 2.12), which include: atorvastatin, cerivastatin, fluvastatin, pravastatin, rosuvastatin, and simvastatin	No/yes
Lifestyle	Alcohol consumption	No/yes
	Body mass index (weight in kg)/(height in m)^2	under/normal weight: <25, overweight: 25-30 obese: ≥30
	Smoking status	No/ex/yes
Demography	Sex	Female/male
	Socioeconomic status measured by Mosaic, which is a consumer classification that captures demographics, lifestyles and behaviour of a person	10 categories, see Table 2
	Year of birth category	1920-25, 1926-29, 1930-35, 1936-40
District	Air pollution, which include separate variables for 2001 estimated level of nitrogen dioxide, nitrogen oxide, sulphur dioxide, and particulate matter	Quintiles
	Ethnicity, which include separate variables for proportion of district population defining themselves as white, mixed, Asian or Asian British, black or black British, and other	Quintiles
	Index of multiple deprivation (IMD)	Quintiles in which 1 is most affluent and 5 most deprived
	Limiting long-term illness	Quintiles
	Urbanisation	Urban; town and fringe; village, hamlet and isolated dwelling
	Acute myocardial infarction cases by initial age in the general practice	Percentage

*Latest reading before entering the study, which was at the 1st of January of the year the participant turned the cohort's age. †First category was the baseline.

Table A2 Mosaic coding on postcode level[10]

Category*	Description
Alpha territory†	Most wealthy and influential individuals
Professional rewards	Executive and managerial classes
Rural solitude	People who live in small villages
Small town diversity	People who live in medium sized and smaller towns
Careers and kids	Young couples, married or living with their partner
New homemakers	Neighbourhoods containing mostly houses that were built in the last five years
Ex-council community	Neighbourhoods populated by people who are practical and enterprising
Claimant cultures	Some of the most disadvantaged people
Upper floor living	People who are on limited incomes
Other	People living in neighbourhoods not mentioned above

*Latest reading before entering the study, which was at the 1st of January of the year the participant turned the cohort's age. †Baseline category.

Table A3 Prevalence antiplatelet therapy in age cohorts

Cohort*	DAPT†	Aspirin only	Other antiplatelet agent only
Age 60	122 (1%)	2,213 (13%)	119 (1%)
Age 65	1,079 (3%)	10,152 (23%)	387 (1%)
Age 70	4,097 (6%)	22,639 (31%)	802 (1%)
Age 75	5,565 (7%)	28,451 (37%)	1,009 (1%)

*The age cohorts included cases with history of acute myocardial infarction who were matched to three controls on sex, year of birth category, and general practice. †DAPT=dual antiplatelet therapy (aspirin plus second antiplatelet agent). The prevalence of treatment by the initial ages was affected by calendar year (Figure A2 in the Online Data Supplements).

Table A4 Characteristics of participants with complete and incomplete* medical records

Cohort†	Records	Year of birth	Size	Annual death rate (/1,000)	AMI (%)
Age 60	Complete	1936-40	6,901	14.9	1,869 (27%)
		1930-35	2,374	19.1	674 (28%)
	Incomplete	1936-40	3,691	14.3	779 (21%)
		1930-35	3,778	18.1	864 (23%)
	Total		16,744	16.4	4,186 (25%)
Age 65	Complete	1936-40	16,357	17.9	4,607 (28%)
		1931-35	9,568	23.0	2,595 (27%)
		1925-30	2,946	29.7	810 (28%)
	Incomplete	1936-40	5,339	15.8	817 (15%)
		1931-35	4,608	22.8	949 (21%)
		1925-30	4,710	30.2	1,104 (23%)
	Total		43,528	22.5	10,882 (25%)
Age 70	Complete	1936-40	20,790	21.6	5,631 (27%)
		1931-35	19,645	27.1	5,486 (28%)
		1920-30	13,718	38.7	3,760 (27%)
	Incomplete	1936-40	2,774	19.4	260 (9%)
		1931-35	6,143	27.1	961 (16%)
		1920-30	10,658	41.2	2,334 (22%)
	Total		73,728	32.5	18,432 (25%)
Age 75	Complete	1931-36	27,648	32.7	7,393 (27%)
		1926-30	20,266	44.8	5,624 (28%)
		1920-25	11,035	59.0	3,016 (27%)
	Incomplete	1931-36	3,240	32.1	329 (10%)
		1926-30	6,854	46.5	1,156 (17%)
		1920-25	7,349	58.0	1,580 (22%)
	Total		76,392	47.6	19,098 (25%)

*Missing record in alcohol consumption status, body mass index, or smoking status. †The age cohorts included cases with history of acute myocardial infarction (AMI) who were matched to three controls on sex, year of birth category, and general practice.

Table A5 Distribution of known and imputed values* of variables with missing data

	**Age 60**		**Age 65**		**Age 70**		**Age 75**	
	**Known**	**Imputed**	**Known**	**Imputed**	**Known**	**Imputed**	**Known**	**Imputed**
Alcohol	84%	84%	82%	80%	80%	79%	77%	75%
BMI (sd)	26.8 (4.2)	26.3 (4.3)	27.0 (4.3)	26.4 (4.3)	27.1 (4.2)	26.2 (4.3)	26.8 (4.4)	25.9 (4.3)
Ex-smoker	20%	19%	27%	25%	33%	32%	35%	34%
Smoker	30%	30%	25%	24%	19%	17%	15%	13%

*mean across ten imputed datasets.

Table A6 Prevalence coronary revascularisation given ischaemic heart disease (IHD)

Cohort*	Coronary revascularisation†	n (%)
Age 60	CABG	751 (16%)
	PCI	167 (3%)
	Total*	881 (18%)
Age 65	CABG	2,479 (19%)
	PCI	750 (6%)
	Total*	3,069 (23%)
Age 70	CABG	4,606 (19%)
	PCI	1,869 (8%)
	Total*	6,113 (26%)
Age 75	CABG	5,036 (19%)
	PCI	1,958 (7%)
	Total*	6,601 (25%)

*The prevalence of coronary revascularisation by the initial ages was affected by calendar year (Figure A2 in the Online Data Supplements). †CABG=coronary artery bypass graft. PCI=percutaneous coronary intervention. Some IHD patients had both CABG and PCI.

Figure A1 Prevalence of history of ischaemic heart disease (IHD) and coronary revascularisation given IHD by deprivation quintiles (IMD)

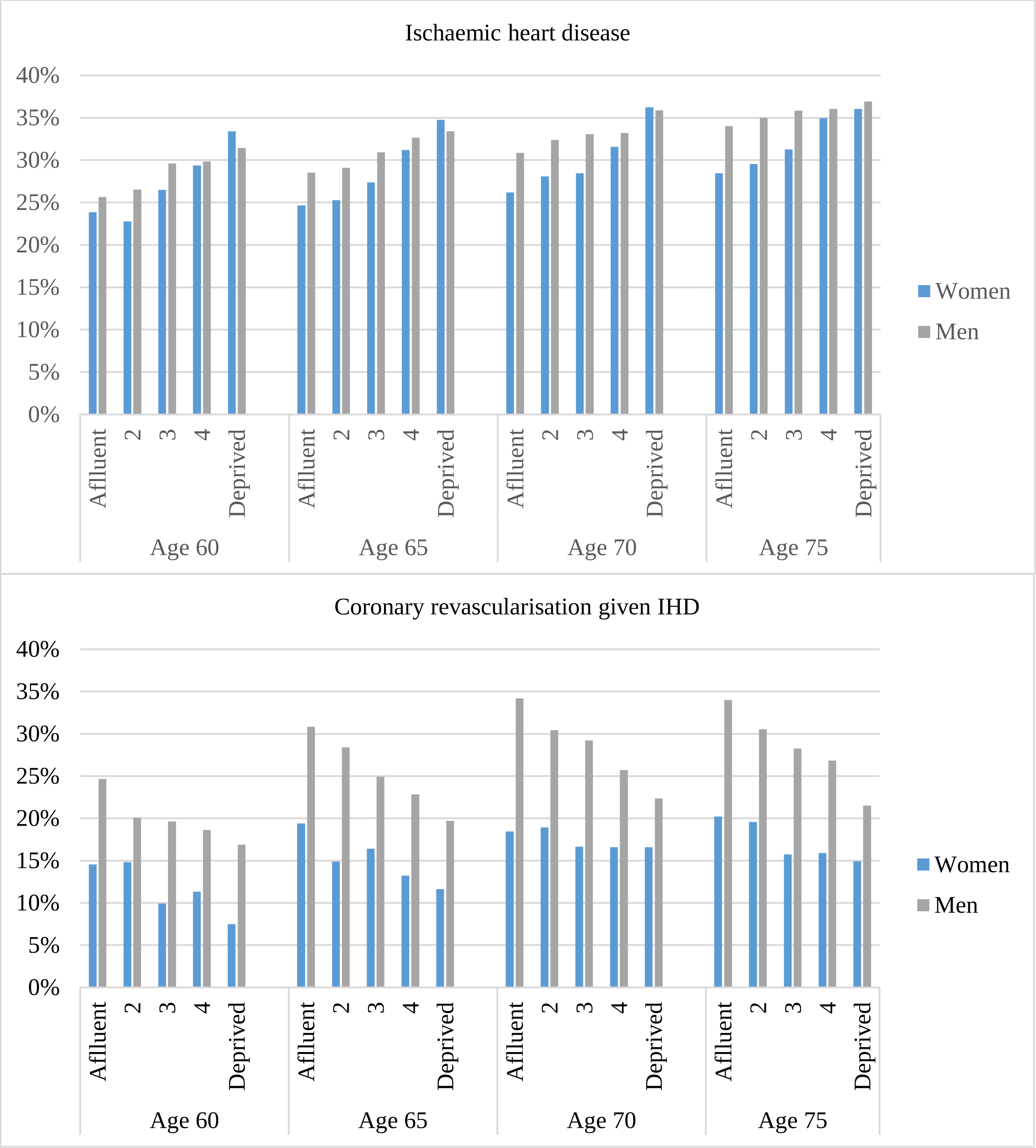

Table A7 Prevalence of diabetes in men and women with a history of ischaemic heart disease

Coronary Revascularisation	Sex	Age 60	Age 65	Age 70	Age 75
No	Women	98 (12%)	375 (15%)	864 (18%)	1,171 (18%)
	Men	315 (10%)	1,093 (14%)	2,209 (17%)	2,518 (19%)
Yes	Women	24 (23%)	80 (19%)	217 (21%)	306 (24%)
	Men	86 (11%)	430 (16%)	1,081 (21%)	1,219 (23%)

Figure A2 Prevalence of treatment by cohort's age in patients with a history of acute myocardial infarction

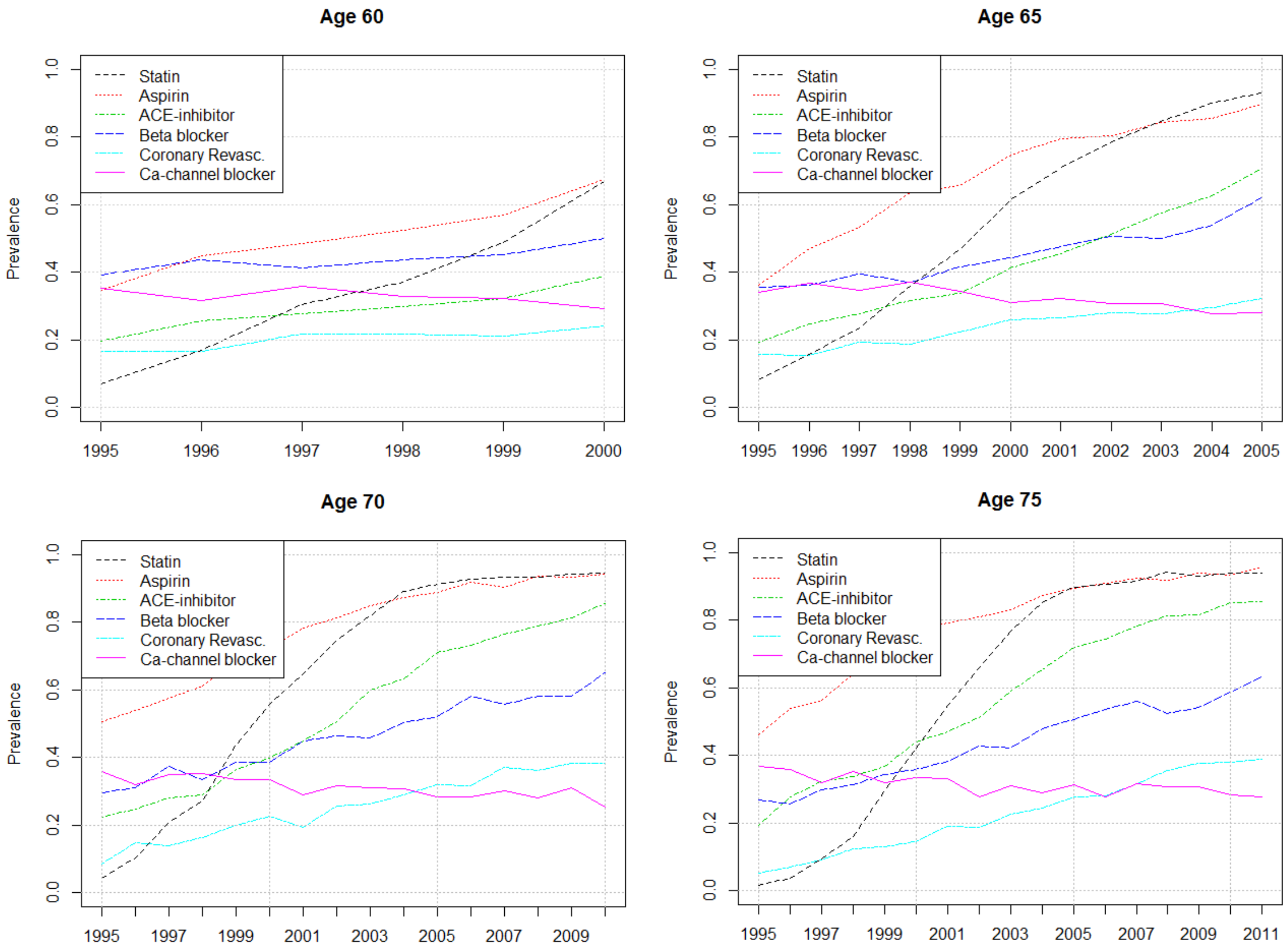

Figure A3 Adjusted survival curves of a history of ischaemic heart disease (IHD)

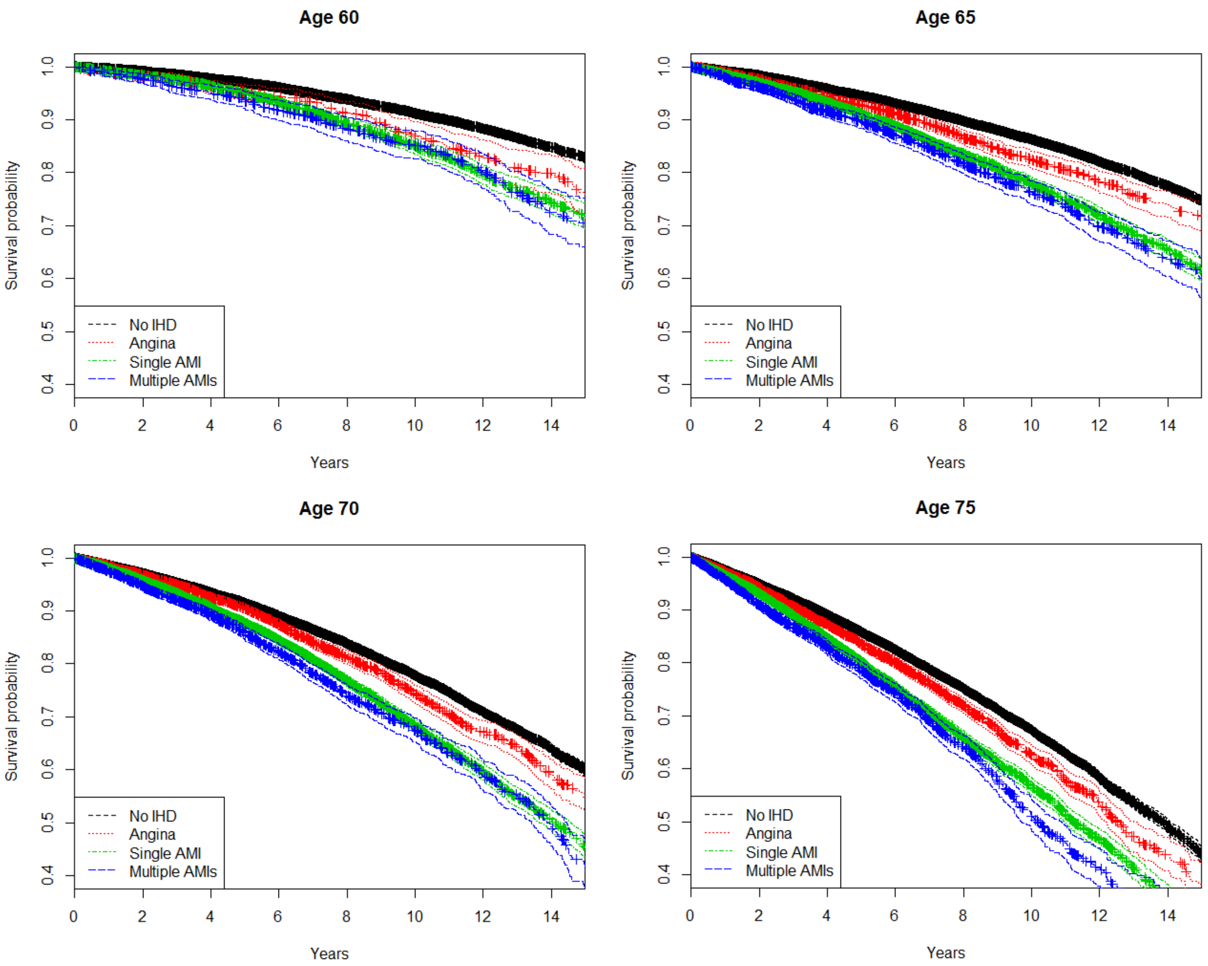

The age cohorts included cases with history of acute myocardial infarction (AMI) who were matched to three controls on sex, year of birth category, and general practice. The hazard of mortality associated with single/multiple AMIs included possible history of angina. The survival curves (confidence intervals) were adjusted for sex, year of birth, socioeconomic status, heart failure, other cardiovascular conditions, chronic kidney disease (only at ages 70 and 75), diabetes, hypertension, hypercholesterolaemia, coronary revascularisation, statins, beta blockers, ACE inhibitors, calcium-channel blockers, aspirin, alcohol consumption, body mass index, smoking status, and general practice.

Figure A4 Survival model at age 60

Factor	Category	Adjusted** HR (95%CI)
Sex	Female	
	Male	1.30 (1.18-1.44)
Year of birth	1936-40	
	1927-35	1.06 (0.98-1.15)
Mosaic	1	
	2	1.14 (0.91-1.44)
	3	1.32 (1.11-1.57)
	4	1.44 (1.20-1.73)
	5	1.72 (1.42-2.09)
	6	1.62 (1.35-1.94)
	7	1.52 (1.23-1.88)
	8	1.67 (1.37-2.02)
	9	1.35 (1.07-1.69)
	10	1.69 (1.38-2.08)
IHD*	No	
	Angina	1.50 (1.25-1.80)
	Single AMI	1.80 (1.60-2.02)
	Multiple AMIs	1.92 (1.60-2.29)
Heart failure	No	
	Yes	1.65 (1.35-2.01)
CVS	No	
	Yes FU<5yrs	2.52 (2.13-2.99)
	Yes FU=5-9yrs	1.82 (1.56-2.13)
	Yes FU>=10yrs	1.42 (1.20-1.68)
Diabetes	No	
	Yes	1.83 (1.62-2.05)
Hypertension	No	
	Yes	1.16 (1.05-1.27)
Cholesterol*	No	
	HCL	0.88 (0.79-0.97)
	Statin	0.81 (0.71-0.93)
Beta blocker	No	
	Yes with AMI	0.83 (0.73-0.94)
	Yes without AMI	0.96 (0.83-1.11)
ACE inhibitor	No	
	Yes	1.10 (0.98-1.24)
Ca-channel blocker	No	
	Yes with AMI	1.04 (0.92-1.18)
	Yes without AMI	1.22 (1.04-1.43)
Aspirin	No	
	Yes	1.10 (0.98-1.22)
Coronary revasc.	No	
	Yes FU<5yrs	0.80 (0.61-1.05)
	Yes FU>=5yrs	0.92 (0.78-1.10)
Alcohol	No	
	Yes	0.93 (0.84-1.04)
BMI&Smoking	NormalW&NS	
	NormalW&ES	1.71 (1.36-2.16)
	NormalW&CS	2.66 (2.25-3.15)
	Overw&NS	1.11 (0.95-1.30)
	Overw&ES	1.52 (1.25-1.84)
	Overw&CS	2.22 (1.84-2.67)
	Obese&NS	1.39 (1.14-1.68)
	Obese&ES	1.79 (1.44-2.22)
	Obese&CS	2.25 (1.84-2.75)

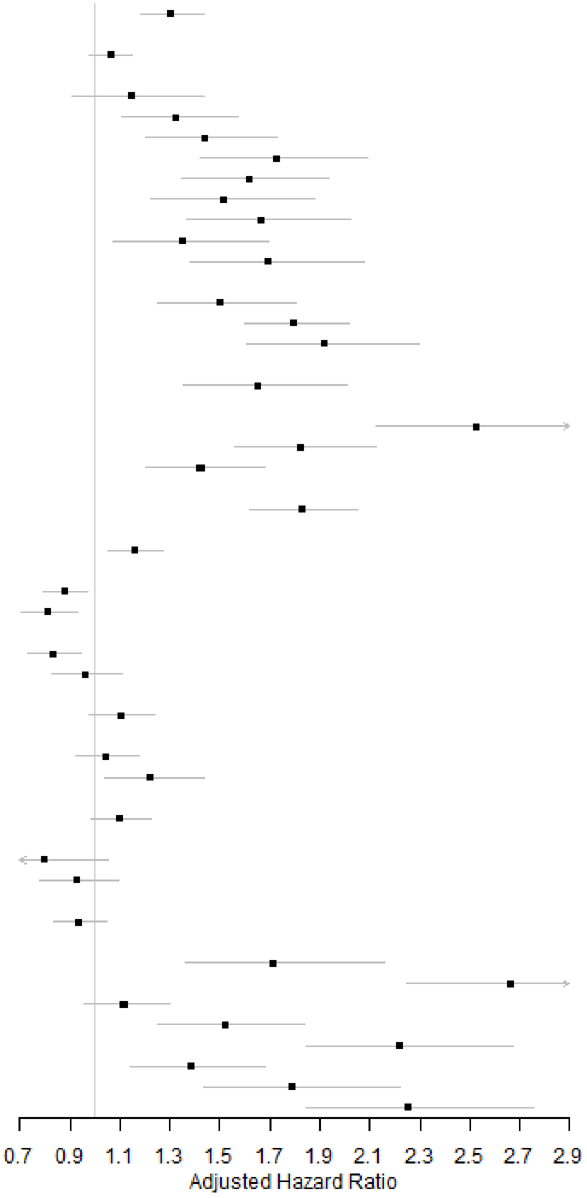

The age cohort included cases with history of acute myocardial infarction (AMI) who were matched to three controls on sex, year of birth category, and general practice. *Hazard ratios (HR) of single and multiple acute myocardial infarction (AMI) were the same with and without angina, and HR of statins was the same with and without hypercholesterolaemia (HCL). **Adjusted for all other factors in model and random effect on general practice.

Figure A5 Survival model at age 65

Factor	Category	Adjusted** HR (95%CI)
Sex	Female	
	Male	1.30 (1.23-1.38)
Year of birth	1936-40	
	1931-35	1.13 (1.06-1.20)
	1922-30	1.33 (1.22-1.44)
Mosaic	1	
	2	1.15 (1.01-1.31)
	3	1.09 (0.99-1.21)
	4	1.25 (1.12-1.39)
	5	1.54 (1.37-1.73)
	6	1.34 (1.21-1.49)
	7	1.44 (1.28-1.63)
	8	1.20 (1.07-1.34)
	9	1.08 (0.94-1.23)
	10	1.40 (1.24-1.58)
IHD*	No	
	Angina	1.21 (1.10-1.34)
	Single AMI	1.71 (1.59-1.84)
	Multiple AMIs	1.87 (1.68-2.07)
Heart failure	No	
	Yes	1.69 (1.52-1.87)
CVS	No	
	Yes FU<5yrs	2.03 (1.87-2.21)
	Yes FU>=5yrs	1.52 (1.41-1.63)
Diabetes	No	
	Yes	1.67 (1.56-1.78)
Hypertension	No	
	Yes	1.00 (0.95-1.06)
Cholesterol*	No	
	HCL	0.85 (0.80-0.91)
	Statin	0.75 (0.70-0.81)
Beta blocker	No	
	Yes with AMI	0.79 (0.73-0.85)
	Yes without AMI	0.98 (0.90-1.06)
ACE inhibitor	No	
	Yes	1.25 (1.17-1.34)
Ca-channel blocker	No	
	Yes with AMI	1.03 (0.96-1.12)
	Yes without AMI	1.27 (1.17-1.37)
Aspirin	No	
	Yes	1.05 (0.99-1.12)
Coronary revasc.	No	
	Yes FU<5yrs	0.72 (0.63-0.82)
	Yes FU>=5yrs	0.95 (0.85-1.06)
Alcohol	No	
	Yes	0.90 (0.84-0.95)
BMI&Smoking	NormalW&NS	
	NormalW&ES	1.52 (1.35-1.72)
	NormalW&CS	2.29 (2.09-2.51)
	Overw&NS	0.96 (0.88-1.04)
	Overw&ES	1.26 (1.15-1.38)
	Overw&CS	1.84 (1.66-2.02)
	Obese&NS	1.10 (0.99-1.23)
	Obese&ES	1.44 (1.29-1.61)
	Obese&CS	1.95 (1.71-2.23)

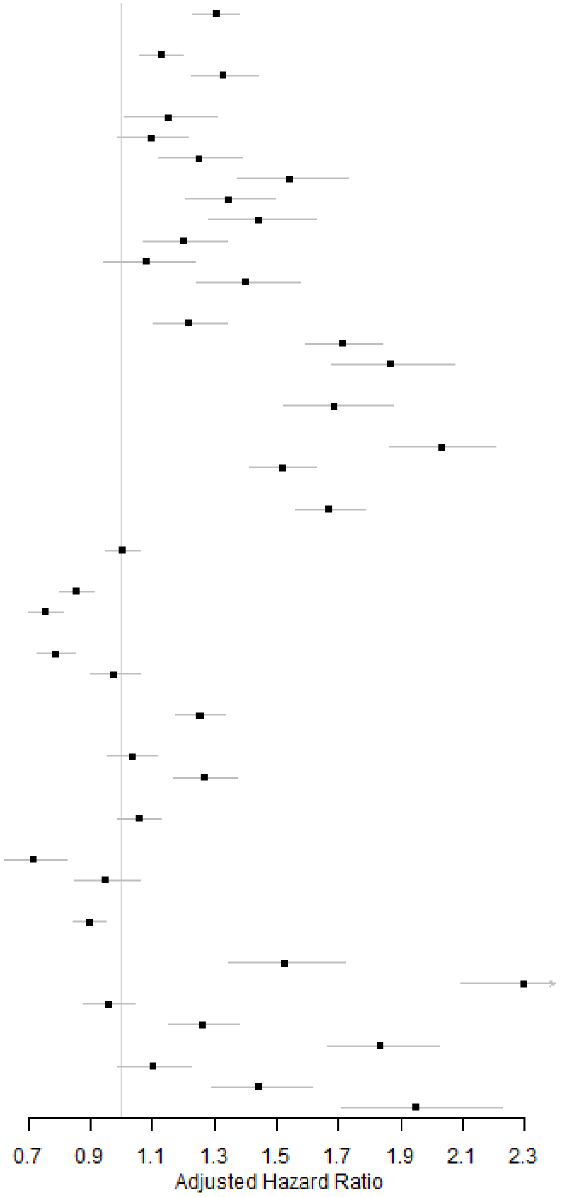

The age cohort included cases with history of acute myocardial infarction (AMI) who were matched to three controls on sex, year of birth category, and general practice. *Hazard ratios (HR) of single and multiple acute myocardial infarction (AMI) were the same with and without angina, and HR of statins was the same with and without hypercholesterolaemia (HCL). **Adjusted for all other factors in model and random effect on general practice.

Figure A6 Survival model at age 70

Factor	Category	Adjusted** HR (95%CI)
Sex	Female	
	Male	1.37 (1.31-1.42)
Year of birth	1936-40	
	1931-35	1.05 (0.98-1.12)
	1920-30	1.16 (1.08-1.24)
Mosaic	1	
	2	1.08 (0.98-1.19)
	3	1.08 (1.01-1.17)
	4	1.28 (1.18-1.38)
	5	1.40 (1.29-1.53)
	6	1.31 (1.21-1.42)
	7	1.33 (1.23-1.45)
	8	1.16 (1.07-1.26)
	9	1.07 (0.97-1.18)
	10	1.30 (1.19-1.42)
IHD*	No	
	Angina	1.15 (1.08-1.23)
	Single AMI	1.50 (1.42-1.59)
	Multiple AMIs	1.66 (1.53-1.80)
Heart failure	No	
	Yes	1.69 (1.57-1.81)
CVS	No	
	Yes FU<5yrs	1.95 (1.85-2.06)
	Yes FU>=5yrs	1.51 (1.43-1.59)
CKD	No	
	Yes	1.51 (1.31-1.74)
Diabetes	No	
	Yes	1.51 (1.44-1.59)
Hypertension	No	
	Yes FU<5yrs	0.95 (0.90-1.00)
	Yes FU>=5yrs	1.03 (0.98-1.09)
Cholesterol*	No	
	HCL	0.86 (0.82-0.91)
	Statin	0.74 (0.70-0.78)
Beta blocker	No	
	Yes with AMI	0.85 (0.81-0.91)
	Yes without AMI	0.96 (0.91-1.02)
ACE inhibitor	No	
	Yes	1.19 (1.14-1.25)
Ca-channel blocker	No	
	Yes with AMI	1.07 (1.00-1.13)
	Yes without AMI	1.20 (1.14-1.27)
Aspirin	No	
	Yes	1.05 (1.01-1.10)
Coronary revasc.	No	
	Yes FU<5yrs	0.73 (0.67-0.80)
	Yes FU>=5yrs	0.86 (0.78-0.94)
Alcohol	No	
	Yes	0.93 (0.89-0.97)
BMI&Smoking	NormalW&NS	
	NormalW&ES	1.47 (1.37-1.58)
	NormalW&CS	2.14 (2.00-2.30)
	Overw&NS	0.94 (0.88-1.01)
	Overw&ES	1.27 (1.18-1.35)
	Overw&CS	1.77 (1.64-1.91)
	Obese&NS	1.10 (1.02-1.19)
	Obese&ES	1.33 (1.22-1.44)
	Obese&CS	1.94 (1.75-2.15)

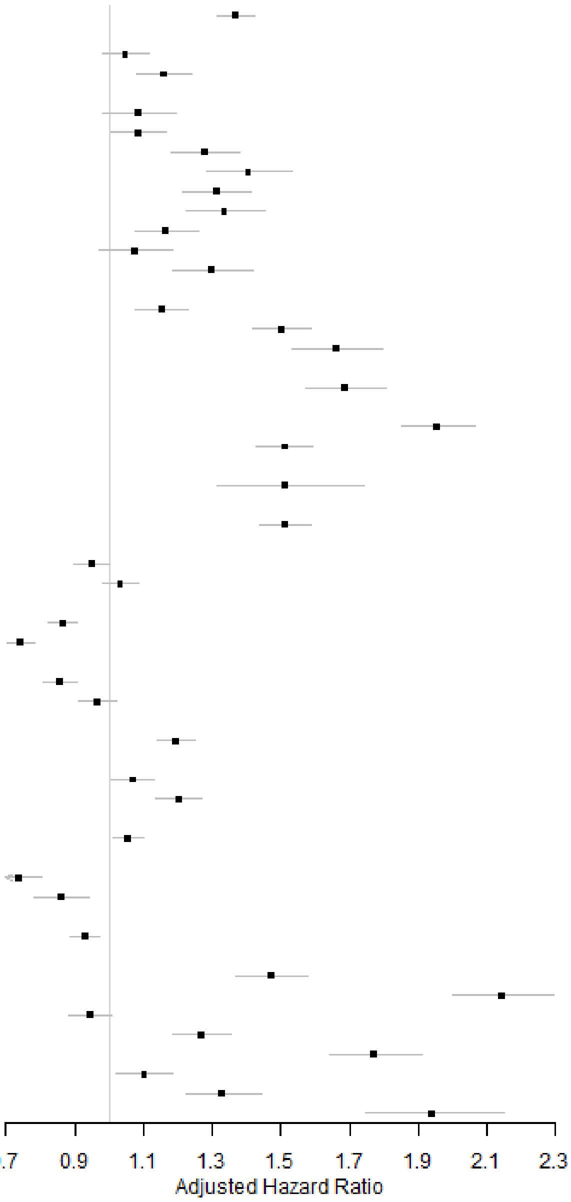

The age cohort included cases with history of acute myocardial infarction (AMI) who were matched to three controls on sex, year of birth category, and general practice. *Hazard ratios (HR) of single and multiple acute myocardial infarction (AMI) were the same with and without angina, and HR of statins was the same with and without hypercholesterolaemia (HCL). **Adjusted for all other factors in model and random effect on general practice.

Figure A7 Survival model at age 75

Factor	Category	Adjusted** HR (95%CI)
Sex	Female	
	Male	1.34 (1.30-1.39)
Year of birth	1931-36	
	1926-30	1.21 (1.14-1.27)
	1920-25	1.29 (1.22-1.37)
Mosaic	1	
	2	1.12 (1.02-1.22)
	3	1.06 (1.00-1.14)
	4	1.20 (1.12-1.29)
	5	1.32 (1.22-1.43)
	6	1.26 (1.17-1.35)
	7	1.26 (1.17-1.36)
	8	1.13 (1.05-1.21)
	9	1.11 (1.02-1.22)
	10	1.20 (1.11-1.30)
IHD*	No	
	Angina	1.16 (1.10-1.22)
	Single AMI	1.45 (1.38-1.53)
	Multiple AMIs	1.63 (1.51-1.76)
Heart failure	No	
	Yes	1.59 (1.50-1.68)
CVS	No	
	Yes FU<5yrs	1.68 (1.61-1.76)
	Yes FU>=5yrs	1.44 (1.36-1.51)
CKD	No	
	Yes	1.40 (1.27-1.55)
Diabetes	No	
	Yes	1.44 (1.38-1.50)
Hypertension	No	
	Yes FU<5yrs	0.94 (0.90-0.98)
	Yes FU>=5yrs	1.06 (1.01-1.11)
Cholesterol*	No	
	HCL	0.85 (0.82-0.89)
	Statin	0.77 (0.74-0.81)
Beta blocker	No	
	Yes with AMI	0.81 (0.77-0.86)
	Yes without AMI	1.00 (0.96-1.05)
ACE inhibitor	No	
	Yes	1.16 (1.11-1.21)
Ca-channel blocker	No	
	Yes with AMI	1.00 (0.95-1.06)
	Yes without AMI	1.12 (1.06-1.17)
Aspirin	No	
	Yes	1.08 (1.04-1.12)
Coronary revasc.	No	
	Yes FU<5yrs	0.78 (0.73-0.84)
	Yes FU>=5yrs	0.97 (0.88-1.06)
Alcohol	No	
	Yes	0.86 (0.82-0.89)
BMI&Smoking	NormalW&NS	
	NormalW&ES	1.49 (1.41-1.58)
	NormalW&CS	2.09 (1.96-2.23)
	Overw&NS	0.96 (0.91-1.02)
	Overw&ES	1.18 (1.12-1.25)
	Overw&CS	1.76 (1.63-1.90)
	Obese&NS	1.05 (0.98-1.13)
	Obese&ES	1.24 (1.15-1.34)
	Obese&CS	1.80 (1.62-1.99)

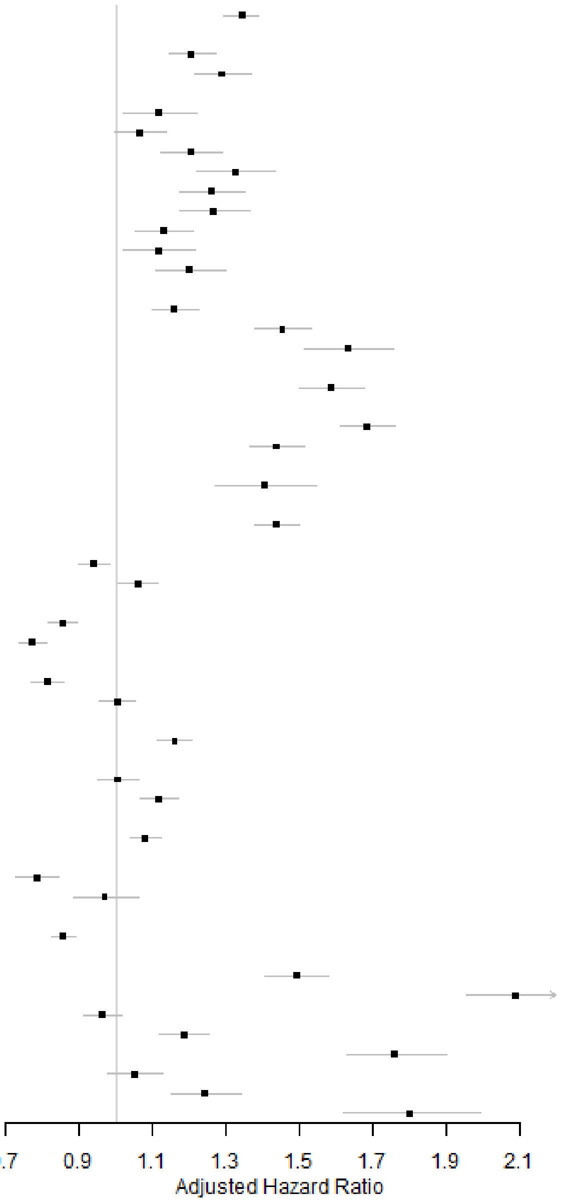

The age cohort included cases with history of acute myocardial infarction (AMI) who were matched to three controls on sex, year of birth category, and general practice. *Hazard ratios (HR) of single and multiple acute myocardial infarction (AMI) were the same with and without angina, and HR of statins was the same with and without hypercholesterolaemia (HCL). **Adjusted for all other factors in model and random effect on general practice.

Table A8 Correlations of districts' characteristics and the adjusted hazards of all-cause mortality associated with general practice

Characteristic	Sub-characteristic	Age 60	Age 65	Age 70	Age 75
Index of multiple deprivation		0.03	0.02	0.10	-0.05
Urbanisation		-0.08	0.03	0.04	0.05
Limiting long-term illness		0.03	0.03	0.10	-0.08
AMI cases by initial ages in general practice		-0.05	-0.04	-0.08	0.02
Ethnicity	White	-0.03	0.01	0.01	0.05
	Mixed	0.00	0.00	0.01	-0.03
	Asian or Asian British	0.01	-0.01	0.00	-0.05
	Black or Black British	0.03	0.01	0.01	-0.07
	Other	0.02	0.02	0.00	-0.04
Air pollution	Nitrogen dioxide	0.00	0.06	-0.01	-0.05
	Nitrogen oxide	0.03	0.07	0.00	-0.08
	Sulphur dioxide	0.08	0.08	-0.01	-0.03
	Particulate matter	0.00	0.06	0.00	-0.05

The age cohorts included cases with history of acute myocardial infarction (AMI) who were matched to three controls on sex, year of birth category, and general practice. The hazards were adjusted for sex, year of birth, socioeconomic status, ischaemic heart disease, heart failure, cardiovascular system conditions, chronic kidney disease (only at ages 70 and 75), diabetes, hypertension, hypercholesterolaemia, coronary revascularisation, statins, beta blockers, ACE inhibitors, calcium-channel blockers, aspirin, alcohol consumption, body mass index, and smoking status. The table reports the Spearman's rank correlations r of districts' characteristics and the adjusted hazards of all-cause mortality associated with general practice, where $|r|=0$ stands for no correlation and $|r|=1$ for perfect correlation between the two variables.[11]

Supplemental References

1. Hennekens CH, Buring JE, Mayrent SL. Epidemiology in medicine. Boston: Little Brown and Company; 1987.
2. Buuren S van. Flexible imputation of missing data. CRC press;2012.
3. Therneau TM. Package 'survival' version 2.37-7. http://r-forge.r-project.org (16 October 2015)
4. Brenner H, Gefeller O, Greenland S. Risk and rate advancement periods as measures of exposure impact on the occurrence of chronic diseases. Epidemiology, 1993;4(3):229-36.
5. Therneau TM, Grambsch PM. Modeling Survival Data: Extending the Cox Model. Statistics for Biology and Health. Springer; 2000.
6. Royston P. Explained variation for survival models. The Stata Journal 2006;6(1):83-96.
7. Steyerberg EW, Vickers AJ, Cook NR, Gerds T, Gonen M, Obuchowski N, Pencina MJ, Kattan MW. Assessing the performance of prediction models: a framework for traditional and novel measures. Epidemiology 2010;21(1):128-138. doi:10.1097/EDE.0b013e3181c30fb2.
8. Harrell FE Jr. Package 'rms' version 4.3-1.http://biostat.mc.vanderbilt.edu/rms (16 October 2015)
9. R [software]. The R Project for Statistical Computing. www.R-project.org (16 October 2015)
10. Experian. Mosaic United Kingdom: the consumer classification of the United Kingdom. http://www.experian.co.uk/assets/business-strategies/brochures/Mosaic_UK_2009_brochure.pdf (16 October 2015)
11. Harrell FE, Lee KL, Mark DB. Tutorial in biostatistics multivariable prognostic models: issues in developing models, evaluating assumptions and adequacy, and measuring and reducing errors. Statistics in Medicine 1996;15:361-387.
